# Characterization of the skin keloid microenvironment

**DOI:** 10.1186/s12964-023-01214-0

**Published:** 2023-08-16

**Authors:** Mengwen Zhang, Hailong Chen, Huan Qian, Chen Wang

**Affiliations:** The Second Affiliated Hospital of Zhejiang University School of Medicine, 88 Jiefang Road, Hangzhou, 310009 China

**Keywords:** Keloid microenvironment, Keloid-derived fibroblasts, Keratinocytes, Vascular endothelial cells, Immune cells, Stem cells, Extracellular matrix, Angiogenesis

## Abstract

Keloids are a fibroproliferative skin disorder that develops in people of all ages. Keloids exhibit some cancer-like behaviors, with similar genetic and epigenetic modifications in the keloid microenvironment. The keloid microenvironment is composed of keratinocytes, fibroblasts, myofibroblasts, vascular endothelial cells, immune cells, stem cells and collagen fibers. Recent advances in the study of keloids have led to novel insights into cellular communication among components of the keloid microenvironment as well as potential therapeutic targets for treating keloids. In this review, we summarized the nature of genetic and epigenetic regulation in keloid-derived fibroblasts, epithelial-to-mesenchymal transition of keratinocytes, immune cell infiltration into keloids, the differentiation of keloid-derived stem cells, endothelial-to-mesenchymal transition of vascular endothelial cells, extracellular matrix synthesis and remodeling, and uncontrolled angiogenesis in keloids with the aim of identifying new targets for therapeutic benefit.

Video Abstract

**Supplementary Information:**

The online version contains supplementary material available at 10.1186/s12964-023-01214-0.

## Background

Keloids are characterized by the excessive deposition of collagen during abnormal wound healing [1], which leads to a fibroproliferative inflammatory response with neovascularization at the leading edge [2]. Life quality of keloid patients is dramatically affected by physical symptoms, including pruritus and pain, as well as psychological barriers [3]. There are currently various treatment options for keloids, and silicone gel with intralesional corticosteroid injections is first recommended. Surgical resection with postoperative radiation or adjuvant pharmacotherapies, radiation therapies, oral medications and laser-based therapies are also used to treat keloids [4–6]. In addition to corticosteroids, injectables also include 5-fluorouracil, botulinum toxin, verapamil, and avotermin. Local radiation therapies include electron beam radiotherapy and low- or high-dose-rate brachytherapy. Pulsed dye lasers include carbon dioxide (CO2) lasers, 585-nm pulsed-dye lasers, and neodymium-doped yttrium aluminum garnet (Nd:YAG) lasers. Unfortunately, there is very low-certainty evidence to support the effectiveness of these laser therapies for treating keloids [7]. As is observed in the clinic, keloids are often refractory to treatment; thus, the treatment of keloids is a significant therapeutic challenge. To identify keloid-specific pathogenetic processes, we aimed to identify more specific therapeutic targets for keloids.

Keloid formation is influenced by various factors, such as wound tension, genetic factors (age, race, family history), hormone levels [8], and lifestyle [9, 10]. Multiple hypotheses for keloid formation have been suggested, such as dysregulation of inflammatory signaling pathways [11, 12], the transforming growth factor β (TGF-β)/small mothers against decapentaplegic (Smad) signaling pathways [13, 14], and the Yes-associated protein (YAP)/transcriptional coactivator with a PDZ-binding domain (TAZ) signaling pathway [15]. Keloids have high infiltration of profibrotic immune cells, such as M2 macrophages and Th2 cells, which promote fibroblast activation via the TGF-β1 signaling pathway [11]. Increased expression of the proinflammatory cytokines IL-6 and IL-8 and decreased expression of the anti-inflammatory cytokine IL-10 promote scarring, which is mediated by the Janus kinase (JAK)/signal transducer and activator of transcription (STAT) signaling pathway [16, 17]. IL-6 can also promote epithelial-mesenchymal transition (EMT) via the JAK/STAT pathway in keloid pathogenesis [18].

**Fig. 1:**
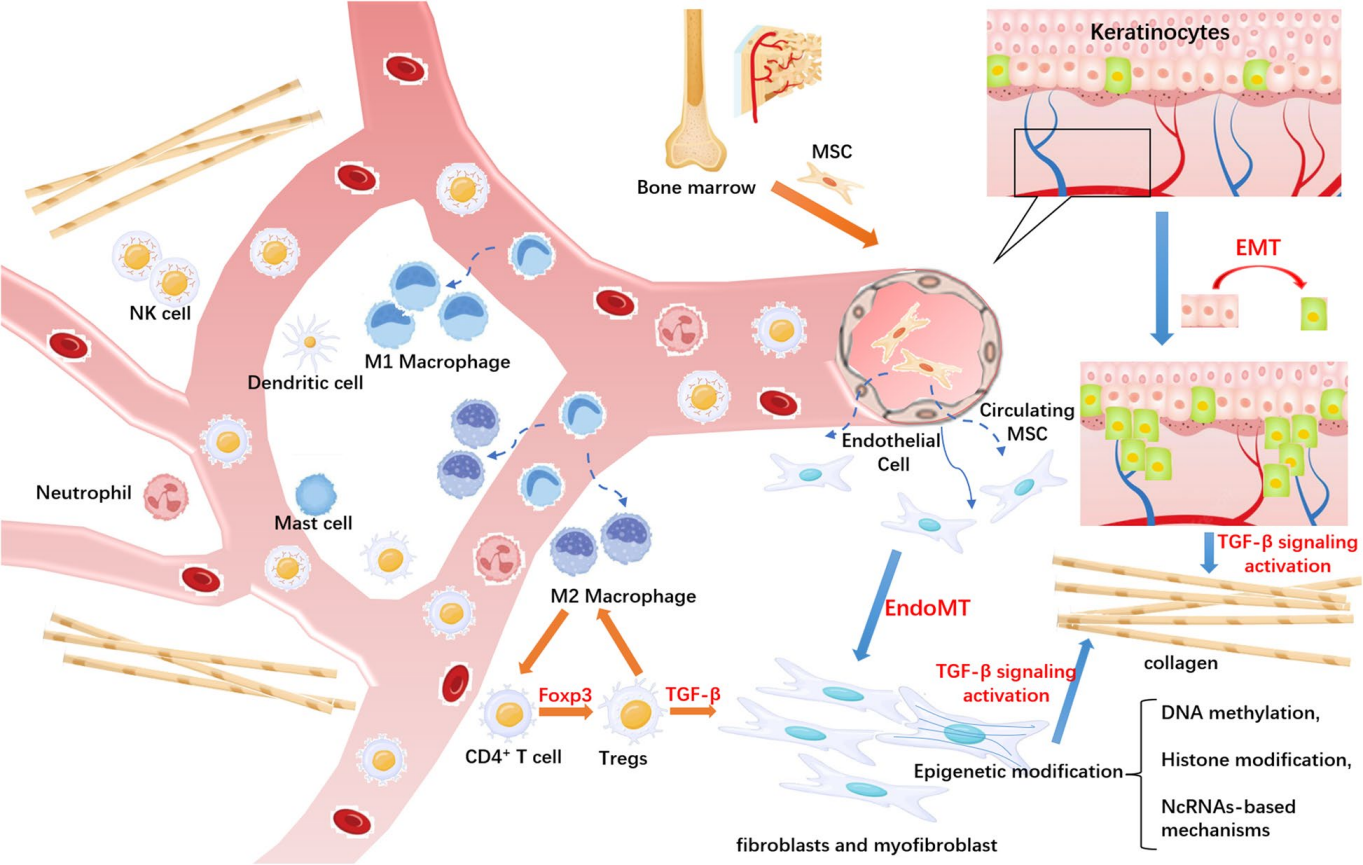
Schematic representation of the keloid microenvironment. The keloid microenvironment includes keratinocytes, fibroblasts, vascular endothelial cells, immune cells, and stem cells with irregularly oriented collagen fibers. Cellular communication in the keloid microenvironment induces the phenotypic and functional reprogramming of KFs, immune cells, and VECs, promoting collagen production via increased TGF-β production

Keloids are classified as a benign fibroproliferative dermal disease, though they exhibit some cancer-like behaviors, such as invasion of neighboring tissues and a high recurrence rate. Various interactions between the components of the keloid microenvironment have been explored, which may explain the aggressive clinical behavior of keloids. Increasing evidence has shown that keloids exhibit cellular bioenergetics, genetic and epigenetic changes and EMT processes that are similar to those in cancers to some extent. The keloid microenvironment includes keratinocytes, fibroblasts, vascular endothelial cells, immune cells, and stem cells with irregularly oriented collagen fibers (Fig. 1). The mechanisms underlying the regulation of the keloid microenvironment have not been fully explored. We discuss the role of stromal cells in the pathogenesis of keloids in this review, which may provide anti-keloid therapeutic approaches.

## Genetic and epigenetic regulation of keloids

Fibroblasts are the main cellular components of the keloid microenvironment, as well as the main effector cells regulating extracellular matrix (ECM) synthesis and remodeling [19]. Some of the cancer-like behaviors of keloids are their continuous activation of fibroblasts and invasive growth into the normal skin adjacent to the original scars [20]. We described the function of fibroblasts, as well as the activated signaling pathways in keloids, in our previous review [21]. As reported, the TGF-β/Smad signaling pathway controls fibroblast differentiation [22], maintaining homeostasis, cell proliferation, migration, and differentiation, and collagen production [13, 14]. Further functional analysis showed that fibroblast differentiation and excessive deposition of ECM are induced by the increased expression of TGF-β1, which is mediated by Dpp4 and urokinase (PLAU) in vitro [23]. TGF-β/Smad signaling pathway activation also maintains hypoxia-inducible factor 1α (HIF-1α) stability [24]. In addition, hypoxia can induce PI3K/Akt and ERK1/2 signaling pathway activation [25], resulting in the accumulation of HIF-1α in keloids, contributing to overactivation of the fibrotic signaling pathway and eventually leading to aggravated fibrosis and infiltrative growth [26]. Gao et al*.* showed increased nuclear YAP/TAZ staining in KFs compared with normal skin [15]. Activation of YAP/TAZ in the nucleus leads to the expression of profibrotic genes, increased α-SMA expression and ECM deposition [27]. KFs also have higher expression of integrin α1β1 and α2β1. Integrin α1β1 promotes fibroblast proliferation but inhibits collagen synthesis, whereas α2β1 has the opposite effect [28]. Moreover, Song et al*.* demonstrated that integrin αvβ3 is more sensitive to mechanical tension and may be a new target for keloid treatment and prevention of keloid recurrence [29].

Increasing evidence suggests that genetic and epigenetic mechanisms strongly contribute to keloid formation [30], and these mechanisms are mainly related to imbalance of fibroblast proliferation and apoptosis, which contributes to the uncontrolled proliferation in keloids. Recently, genomic susceptibility loci related to keloid formation have been identified by genome-wide association studies (GWAS), and single nucleotide polymorphisms (SNPs) were the most common genomic susceptibility loci [31–33]. NEDD4 was recognized as a potentially common genetic element that contributes to keloid development in African American, Japanese, Egyptian and Chinese populations [34–37]. NEDD4 promotes fibroblast proliferation and invasiveness by activating TGF/β-catenin transcriptional activity and NF-κB signaling [38, 39]. A GWAS was conducted in a large Chinese family with a history of keloids, and the results showed that the loci 15q22.31-q23, 18q21.1 and 10q23.31 were related to keloids [40]. Notably, the 18q21.1 locus has been proposed to contain the Smad2, Smad4, and Smad7 genes, which are downstream effectors of the TGF-β signaling pathway. Moreover, the 18q21.1 locus encodes a protein inhibitor of the PIAS2 gene [41], which has a negative effect on mitochondrial homeostasis and oxidative stress in cells [42].

Epigenetics includes DNA methylation, histone modification, and ncRNA-based mechanisms [43]. Epigenetic changes are crucial for the activation of KFs. Alghamdi et al*.* performed genome-wide scanning of methylated CpG sites in 12 KFs and 12 control skin fibroblasts (CFs) and identified 100,000 differentially methylated CpG sites, more than half of which were hypermethylated [44]. The most functionally enriched genes were significantly associated with the regulation of transcription, DNA templating, and the nucleus, revealing a potential mechanism underlying keloid formation. Stevenson et al*.* performed both expression and methylation array analyses with normotrophic scar fibroblasts and CFs, and the combined analysis showed that FOXF2 was both significantly differentially methylated and expressed in normotrophic scars. Further analysis showed that FOXF2 knockdown significantly reduced collagen I production in KFs. These results suggest that FOXF2 has an important effect on scar maintenance [45]. FOXF2 has been implicated in EMT in skin and forms a positive feedback loop with TGF-β signaling [46, 47]. Hu et al*.* reported that DNA methyltransferase (DNMT)-mediated DNA methylation regulates α-SMA gene expression during myofibroblast differentiation [48]. Niu et al*.* found that overexpression of DNMT3A inhibited TGF-β expression by decreasing 5hmC, which is located in the TGF-β promoter region, thus inhibiting fibroblast proliferation [49]. Recently, increasing attention has been given to m^6^A methylation, which is a posttranscriptional modification of eukaryotic mRNAs and lncRNAs [50]. m^6^A methylation directly controls gene expression by reversing methylation by fat mass and obesity-associated protein (FTO) and AlkB homolog 5 (ALKBH5) [51, 52]. Lin et al*.* performed m^6^A sequencing and RNA sequencing to compare m^6^A-modified RNA patterns between normal skin and keloid tissue. The results showed that more unique m^6^A methylation peaks and m^6^A-related gene transcripts were identified in keloid samples, and these genes were mainly related to the Wnt signaling pathway. These findings further confirmed that m^6^A methylation activation and Wnt/β-catenin signaling activation exist in keloids [53]. Furthermore, Xie et al*.* analyzed two public datasets (GSE44270 and GSE145725) and established a keloid risk prediction model. The gene expression of reported m^6^A-related genes was compared between high-risk and low-risk groups to explore the connection between m^6^A methylation and keloid risks. The results showed that ALKBH5, FTO, and HNRNPA2B1 were highly expressed while YTHDF2 was expressed at low levels in the high-risk group [54]. This study highlights the importance of developing individualized therapy according to clinical risk grouping.

Histone modifications can affect genome function directly or indirectly by various posttranslational regulatory mechanisms [55]. Research on histone modification in keloids has mainly focused on inhibiting histone deacetylases (HDACs), which remove acetyl groups from histone tails to mediate histone modification. KFs overexpress HDAC2, and the HDAC inhibitor trichostatin A (TSA) can inhibit TGF-β1-induced collagen synthesis in KFs [56]. Another study showed that the expression of fibrosis-associated genes, including SFRP1, insulin-like growth factor binding protein 5 (IGFBP5), collagen 1 (COL1) and CTGF, was decreased in KFs after treatment with TSA [57]. Hsu et al*.* also confirmed that TSA can decrease the expression of fibrosis-associated genes in keloids, including RUNX family transcription factor 2 (RUNX2) and fibronectin [58]. Another HDAC inhibitor, CUDC‑907, reversed the pathological phenotype of KFs by inhibiting HDAC2 and PI3K/Akt/mTOR signaling [59]. There is currently little knowledge of histone modifications in keloids, and most studies employ cultured KFs, with little information about the effect on the keloid microenvironment.

Most genomic DNA does not encode proteins but is transcribed into ncRNAs, including microRNAs (miRNAs), long noncoding RNAs (lncRNAs), and circular RNAs (circRNAs). NcRNAs regulate gene expression via the transcriptional and posttranscriptional control of target genes [60]. NcRNA expression profiles were found to be altered in KFs by high-throughput sequencing and gene microarray analyses, regulating various signaling pathways related to the formation and progression of keloids. Recently, an increasing number of ncRNAs have been further studied to elucidate their functions in keloids (Table 1).

**Table 1:** ncRNAs in keloid fibroblasts

miRNA	Expression	Targets	Mechanism
miRNA-21	Up	Smad7 [61];	Promote collagen production
miRNA-21-5p	Up	PTEN [62];	Promote KF autophagy and migration
miRNA-152-3p	Up	FOXF1 [63];	Promote cell proliferation and ECM-related protein expression
miRNA-31	Up	HIF1AN [64];	Promote KF proliferation and decrease KF apoptosis
miRNA-203	Down	EGR1 and FGF2 [65]	Inhibit KF proliferation, invasion, and ECM production
miRNA-205	Down	VEGF [66]	Inhibit the PI3K/Akt pathway
miRNA-637	Down	Smad3 [67]	Inhibit KF proliferation and metastasis
miRNA-29a	Down	TGF-β1 [68]	Decrease type I and type III collagen mRNA and protein levels
LncRNA	**Expression**	**Targets**	**Mechanism**
CACNA1G-AS1	Up	miR-205 [69]	Suppress miR-205 expression to promote keloid progression
HOXA11-AS	Up	miR-148b-3p [70]	Abrogate the inhibition of IGFBP5 mRNA by targeting miR-148b-3p
LINC01116	Up	miR-203 [71]	Promote keloid progression via miR-203/SMAD5 axis
LINC00937	Down	miR-28-5p [72]	Act as an miR-28-5p sponge, promote MC1R expression
LncRNA H19	Up	miR-196b-5p; miR-214-5p [73]	Promote SMAD5 expression, enhance anaerobic glycolysis of KFs
CircRNA	**Expression**	**Targets**	**Mechanism**
CircNRIP1	Up	FXR1 [74]	Promote miRNA‑503 maturation
CircPTPN12	Up	miRNA-21-5p [75]	Activate Wnt signaling pathway
Circ_101238	Up	miR-138-5p [76]	Promote keloid progression via the miR-138-5p/CDK6 axis
circCOL5A1	Up	miR-877-5p [77] miR-7-5p [78]	Inhibit the miR-877-5p/EGR1 axis, activate the PI3K/Akt signaling pathway
CircPDE7B	Up	miR-661 [79]	Promote keloid progression by the circPDE7B/miR-661/FGF2 pathway
Circ_0057452	Up	miR-7-5p [80] miR-1225-3p [81]	Promote keloid progression via the miR-7-5p/GAB1 axis; promote keloid progression by targeting miR-1225-3p and regulating AFF4 levels

MiRNA-21 is one of the most well-studied ncRNAs; it was first considered an oncogene that promotes tumor cell proliferation and migration and inhibits apoptosis [82]. The same phenomenon has been observed in keloids; KFs secreted more exosomal miRNA-21 than normal skin fibroblasts, and exosomal miRNA-21 promoted the proliferation and collagen production of KFs by inhibiting Smad7 [61, 83]. Shi et al*.* demonstrated that miRNA-203 was downregulated in human KFs, decreasing the proliferation, invasion, and ECM production of KFs by directly repressing EGR1 and FGF2 expression [65]. MiRNAs regulate skin fibrosis by affecting the expression of target genes involved in the activation or inhibition of profibrotic signaling pathways, such as the TGF-beta/Smad3 signaling pathway, the PI3K/Akt/mammalian target of rapamycin (mTOR) signaling pathway, and the Wnt/beta catenin signaling pathway [75]. MiRNAs interact with the 3’ untranslated region (UTR) of target mRNAs, thus regulating target gene expression [84]. He et al*.* identified miRNA-29a-3p as a potential biomarker for keloid treatment through mRNA‒miRNA network analysis, as it was associated with the progression of keloids [85]. An analysis with a pathway-focused lncRNA microarray identified four lncRNA biomarkers in keloids involved in the Wnt pathway [86]. The potential role of HOXA11-AS in KFs was further explored, and HOXA11-AS promotes keloid progression via the miR-148b-3p/IGFBP5 axis and miR-124-3p/TGFβR1 axis [70, 87]. Zhang et al*.* explored the expression profile of circRNAs in human KFs and NFs by bioinformatics analyses and high-throughput RNA sequencing, creating a circRNA-miRNA‒mRNA interaction network by bioinformatics tools [88]. NcRNAs are relatively stable, and they can be easily detected in serum and plasma, suggesting that ncRNAs may be valuable biomarkers for monitoring disease progression and response to treatment.

## EMT in keloids

Stationary epithelial cells lose cell‒cell adhesion and apical‒basal polarity, acquiring mesenchymal characteristics with migratory capacity; this process is called EMT [89]. EMT is the key mechanism underlying organ fibrosis, including fibrosis in the skin [90]. During EMT, cell surface marker expression patterns change from favoring the expression of the epithelial adhesion molecule E-cadherin (CDH1) to favoring the expression of N-cadherin (CDH2) or OB-cadherin (CDH11); mesenchymal cytoskeletal proteins, such as vimentin and β-catenin, are upregulated. Increased cytoplasmic expression and nuclear translocation of β-catenin activate transcription factors and downstream signaling pathways. Hahn et al*.* analyzed the gene expression of keloid keratinocytes and found that many EMT-related genes exhibited aberrant expression, suggesting that EMT is involved in keloid progression [91]. The researchers further showed that the EMT-related gene expression pattern of keloid keratinocytes was regulated by TGF-β1 signaling pathways [90]. TGF-β1 induced EMT in keloid keratinocytes, which was inhibited by the SMAD2/SMAD3 inhibitor SB525334 and ERK 1/2/p38 inhibitor U0126 [92]. They also showed that pirfenidone, a small molecule candidate inhibiting lung fibrosis, inhibited EMT in keloid keratinocytes as well as the migration and proliferation of keloid keratinocytes [93]. Another study demonstrated that the hypoxic microenvironment provides a favorable environment for keloid keratinocytes to adopt a fibroblast-like appearance through EMT, enhancing the invasive capacity of keloid keratinocytes and allowing keloids to extend beyond the wound margin [94].

Fibroblasts are considered to be the result of EMT, which is a hallmark of fibrotic diseases, including keloids [95]. Most published studies have focused on exogenous and endogenous factors that induce EMT in keloid keratinocytes, while KFs and macrophages that differentiate into fibroblasts in the keloid microenvironment have rarely been discussed. Lei et al*.* indicated that hypoxia induced the expression of the EMT marker vimentin and decreased the levels of E-cadherin in KFs, while metformin abolished hypoxia-induced EMT in KFs by inhibiting the HIF-1α/PKM2 signaling pathway [96]. A recent study showed that macrophages can also differentiate into myofibroblasts via a process called the macrophage-to-myofibroblast transition and that two-thirds of fibroblasts in cutaneous punch wounds are derived from cells of myeloid origin [97].

## Immune response in keloid formation

Previous research has mainly focused on the abnormal proliferation of KFs and excessive accumulation of collagens, with little emphasis on immunity. Evidence has shown that keloids are associated with abnormal inflammatory responses to skin injury and exhibit similar features to the skin of patients with autoimmune diseases, such as immune cell infiltration and complement deposition [98]. Studies have shown that collagen synthesis and remodeling are affected by inflammation, and the final scar size is positively correlated with the severity of skin inflammation [12]. A prolonged inflammatory response postwounding is crucial for the development of keloids [12, 99]. Tredget et al*.* showed that a Th2-polarized immune response to injury leads to high expression of fibrogenic cytokines (IL-4 and IL-10) and TGF-β, which in turn promote fibrogenesis [100]. Wu et al*.* analyzed gene and protein expression by RNA-seq and immunohistochemistry analyses of keloid tissues and adjacent normal tissues from African American keloid patients, with healthy skin from African Americans as controls [101]. Their results showed that keloid tissue had significant upregulation of genes related to T lymphocyte activation, Th2 immune response markers, Th1 immune response markers, and Th17/Th22 immune response. Additionally, increased immune cell infiltration was observed in keloid tissues, including infiltration of T lymphocytes, dendritic cells (DCs), and mast cells. A mechanistic study showed that IL-6 maintains the chronic profibrotic state via a Th1-mediated immune response in keloids, and the IL-17/IL-6 axis is also dysregulated [102, 103].

Immune cells in the keloid microenvironment include macrophages, T lymphocytes, B lymphocytes, mast cells, neutrophils, NK cells, and DCs. The roles of these cells in the development of keloids have been separately reported. In particular, macrophages, T cells, and mast cells are increased in keloid tissues [104]. In this section, we discuss how these cells regulate fibrotic processes.

Macrophages are the most widely studied immune cells in tissue remodeling during wound healing. Macrophages can transition into two extreme states when activated by different cytokines: classically activated (M1) macrophages and alternatively activated (M2) macrophages [105]. M1 macrophages are thought to be important and to be present at a higher proportion in the early stage of wound healing, while M2 macrophages are associated with tissue repair and fibrosis and secrete anti-inflammatory cytokines [106]. In the tissue remodeling stage, the number of infiltrating M2 macrophages around the wound is increased, mainly because of recruitment of monocytes from bone marrow and the repolarization of tissue-resident M1 macrophages [107–109]. M2 macrophages promote keloid formation in various ways, such as paracrine signaling and cell-to-cell interactions, and create an anti-inflammatory microenvironment [104, 110]. Li et al*.* showed that the number of infiltrating M2 macrophages was significantly greater than that of infiltrating M1 macrophages in the dermis of keloids [111]. In a prospective study, researchers investigated the baseline numbers of M2 macrophages in the local wound healing microenvironment immediately after incision. Patients who developed HTSs had higher baseline M2 macrophage numbers than patients who had normal scars [112]. M2 macrophage marker genes are upregulated in keloid tissue, and M2 macrophage-derived TGF-β and platelet-derived growth factor–CC (PDGF-CC) are highly related to tissue repair and ECM remodeling [108, 113]. M2 macrophages are not homogeneous, and they are categorized into four subgroups (M2a, M2b, M2c, and M2d) according to their activation signals and biological functions [114]. For example, M2a macrophages are activated by IL-4 and IL-13 and promote fibroblast proliferation, ECM formation, and angiogenesis. M2b macrophages are activated by IL-1 receptor ligands or Toll-like receptors (TLRs) and suppress inflammation via IL-10 signaling. M2c macrophages are activated by IL-10 and TGF-β, remodeling the ECM. M2d macrophages are activated by IL-6 and adenosine, inhibiting M1 macrophages. This M2 macrophage diversity suggests that keloids may be highly infiltrated with M2a macrophages, a combination of macrophage subsets, or even macrophages of an unknown phenotype. Changing the polarization status of macrophages in the keloid microenvironment may be a novel strategy for keloid clinical treatment.

The roles of T cells in the pathological mechanisms of keloid progression have recently been reported by several studies [101, 115]. Analysis of immune cell infiltration into keloid tissues showed that changes in differentially expressed genes (DEGs) were mainly concentrated in T-cell subtypes, followed by NK cells [116]. Foxp3 expression was significantly increased in CD4^+^ T cells after incubation with keloid-derived macrophages. Foxp3 is considered to be a reliable Treg marker, and macrophages promote Treg differentiation by upregulating Foxp3 expression [109]. Tregs function as immunosuppressive cells by inhibiting the activation of CD4^+^ and CD8^+^ T cells while promoting M2 macrophage polarization [117]. Treg cells express TGF-β, which increases the expression of COL1A1 and COL3A1, inducing the overexpression of collagen [115]. Memory T cells in keloids are prone to generating IFN-γ rather than producing TNF-α. FOXP3^+^ CD8^−^ memory T cells have decreased IL-10 secretion, resulting in overexuberant but dysregulated T-cell responses in keloids [118]. DCs are also involved in the formation and progression of keloids [101]. Onodera et al*.* showed that FXIIIa-positive dermal DC infiltration in keloid tissue was significantly higher than that in mature scars [119]. The role of NK cells in keloid formation remains to be further studied in the future.

## Differentiation of keloid-derived stem cells

Stem cells have unlimited self-renewal capacity and can differentiate into various progeny cells with different functions. Increasing evidence has shown that stem cells are involved in the pathogenesis of keloids [120, 121]. Aberrantly activated fibroblasts and myofibroblasts may develop from mesenchymal stem cells (MSCs) through the EMT process [122]. MSCs are identified by the coexpression of unique cell surface markers (CD73, CD90, and CD105) and the lack of endothelial surface markers and common leukocyte antigens such as CD45 and CD34 [123]. Zhang et al*.* successfully reprogrammed KFs into iPSCs in vitro, and this reprogramming may provide a basis for the clinical treatment of keloids. Moon et al*.* identified stem cells that behaved like MSCs with multidifferentiation potential in keloids, and these cells were named keloid-derived mesenchymal-like stem cells (KMLSCs) [124]. Another study also identified a new population of stem cells in keloids that exhibit clonogenicity and self-renewal ability and express MSC surface markers, and these cells are named keloid-derived precursor cells (KPCs). The researchers further reported that the alteration of stem cells was regulated by stimuli from the microenvironmental niche, mediated by an autocrine/paracrine cytokine (IL-17/IL-6) axis.

## Endothelial-to-mesenchymal transition of vascular endothelial cells in keloids

The role of vascular endothelial cells (VECs) in cutaneous wound healing and scar formation has been reported in the literature. Endothelial function is significantly different between patients with keloids and nonkeloid control subjects, and keloid patients are more likely to have poor reactive hyperemia index and augmentation index values [125]. VECs can lose their original adhesive properties and apical‒basal polarity, becoming migratory, spindle-shaped, undifferentiated mesenchymal cells that invade adjacent tissues. This transdifferentiation process is called endothelial-to-mesenchymal transition (EndoMT) [126]. Some keloid fibroblasts and myofibroblasts develop from VECs via EndoMT. Lee et al*.* showed that approximately half of excised keloids (5/12) exhibited characteristics of early-stage EndoMT, which was evaluated by the expression of the mesenchymal marker vimentin in CD31^+^ VECs in the dermal vasculature of keloids. The EndoMT of human dermal VECs can be driven by Wnt-3a in vitro, which plays a critical role in fibrosis [122]. Matsumoto et al*.* investigated gene expression patterns in live VECs isolated from keloid tissues by microarray analysis, and the results showed that 15 genes were upregulated and 3 genes were downregulated in keloid VECs compared with normal VECs. SERPINA3 and LAMC2 were the most upregulated genes in keloid VECs, and they contribute to fibrosis and inflammation in keloids [127]. Tanaka et al*.* compared the number of circulating CD34^+^ cells and their vasculogenic capacity, as well as secretory function, between keloid patients and healthy individuals; the results indicated that CD34^+^ cells derived from keloid patients demonstrated higher expression of Il-8 and VEGF [128]. Single-cell RNA sequencing was performed by two independent teams, and they both observed significant expansion of KF and VEC subpopulations in keloids [129, 130]. In addition, Shim et al*.* used spatial transcriptomics to study the cell‒cell interactions between fibroblasts and endothelial cells in keloids. The results showed that KFs were enriched in the deeper keloid areas, mostly located around the vasculature. Mesenchymal activation was observed in keloid VECs, which was characterized by dysregulation of TGF-β/SMAD signaling. Multiplex immunofluorescence results showed the colocalization of mesenchymal and vascular markers, suggesting the mesenchymal activation of keloid VECs.

## ECM in keloids

The ECM is a complex structure that surrounds and anchors cellular components in tissues. Histopathologic examination of keloids demonstrated that the ECM components, particularly collagens I and III, were arranged in a parallel fashion, accompanied by fibroblasts and myofibroblasts, which are the primary sources of ECM [131]. The ECM not only serves as a structural scaffold and provides mechanical stability for the keloid microenvironment, but also regulates multiple biological functions by presenting cytokines and transmitting signals intracellularly. The biological signals involved in ECM synthesis and deposition are discussed in the first section. We mainly focused on the expression of ECM molecules in the keloid microenvironment. The collagen production in keloids was 20-fold higher than that in normal scars, and the ratio of type I/type III collagen (17:1) in keloids was 3 times higher than that in normal scars (6:1) [132]. It has been reported that the keloid matrix lacks elastin fibers, hyaluronic acid, and dermatopontin, leading to stiffness in keloid tissues [133, 134]. Increased expression of tenascin C was observed during keloid formation in association with collagen fibrils in the reticular dermis, whereas tenascin C was only expressed beneath the basal lamina in normal skin. Increased tenascin C expression has also been reported in acne scarring [135]. Platelet-derived growth factor (PDGF), fibroblast growth factor β (FGF-β), and insulin-like growth factor I (IGF-I) also drive the inflammatory response and excessive ECM deposition [116].

## Excessive angiogenesis during keloid formation

Angiogenesis is necessary in the healing process after injury to promote tissue regeneration. The microvessel density during the wound healing process is 3–10 times that found in normal tissue [136]. Neovascularization is critical for transporting immune cells, oxygen, cytokines and nutrients to the microenvironment in wound healing, and excessive angiogenesis allows abnormal scar healing and keloid formation. Angiogenesis is triggered by hypoxia and several cellular cytokines, including VEGF, PDGF, FGF-β, and members of the TGF-β family [137, 138]. Ogawa et al*.* proposed the hypotheses that dysregulated endothelial function increases immune cell infiltration and that inflammatory cytokines enter the ECM from microvessels, thereby increasing local inflammation and promoting keloid formation [139]. VECs isolated from porcine hypertrophic scars were less permeable than normal VECs, and this increased permeability may contribute to the dysregulated endothelial function of micro blood vessels. In addition, VECs isolated from porcine hypertrophic scars had higher expression of angiogenic genes, such as endothelin-1, angiopoietin-1, and angiopoietin-2, than normal VECs [140]. The cytokines released from the micro blood vessels promote fibroblast proliferation and transition into myofibroblasts, and myofibroblasts produce microvesicles to increase endothelial cell proliferation, migration and assembly into capillary-like structures in return [141, 142]. Thus, abnormal blood vessel regulation plays a role in keloid and hypertrophic scar pathogenesis, and inhibiting abnormal angiogenesis may be an important therapeutic approach.

## Discussion

The keloid microenvironment exhibits cellular heterogeneity and is mainly composed of KFs, myofibroblasts, keratinocytes, keloid-derived stem cells, VECs and immune cells, including macrophages, T lymphocytes, B lymphocytes, mast cells, neutrophils, NK cells and DCs. Each component promotes keloid progression in different ways. Collagen synthesis and excessive ECM deposition are enhanced by genetic and epigenetic changes in KFs and myofibroblasts. Immune cells promote KF proliferation by secreting profibrotic cytokines, VECs and keloid stem cells differentiate into KFs and myofibroblasts via EndoMT, and keratinocytes undergo EMT with increased TGF-β production. Understanding the cellular communication among each component of the keloid microenvironment and the complex signaling pathways involved in the regulation of aberrant ECM deposition will lead to novel treatments for keloids.

## Data Availability

All data generated or analyzed during this study are included in this published article.

## Abbreviations

TGF-β: Transforming growth factor β
Smad: Small mothers against decapentaplegic
EMT: Epithelial-to-mesenchymal transition
KFs: Keloid-derived fibroblasts
CFs: Control skin fibroblasts
YAP: Yes-associated protein
TAZ: Transcriptional coactivator with a PDZ-binding domain
JAK: Janus kinase
STAT: Signal transducer and activator of transcription
HIF-1α: Hypoxia-inducible factor 1α
DNMT: DNA methyltransferase
FTO: Fat mass and obesity-associated protein
HDACs: Histone deacetylases
TSA: HDAC inhibitor trichostatin A
miRNAs: Micrornas
lncRNAs: Long noncoding RNAs
circRNAs: Circular RNAs
DCs: Dendritic cells
EndoMT: Endothelial-to-mesenchymal transition
VECs: Vascular endothelial cells
KPCs: Keloid-derived precursor cells
KMLSCs: Keloid-derived mesenchymal-like stem cells
MSCs: Mesenchymal stem cells
DEGs: Differentially expressed genes
